# Land use change effects on diversity of soil bacterial, Acidobacterial and fungal communities in wetlands of the Sanjiang Plain, northeastern China

**DOI:** 10.1038/s41598-019-55063-4

**Published:** 2019-12-06

**Authors:** Xin Sui, Rongtao Zhang, Beat Frey, Libin Yang, Mai-He Li, Hongwei Ni

**Affiliations:** 1 Heilongjiang Provincial Key Laboratory of Ecological Restoration and Resource Utilization for Cold Region, School of Life Sciences, Heilongjiang University, Harbin, 150080 China; 2 Institute of Nature of Ecology, Heilongjiang Academy of Science, Harbin, China; 3 Swiss Federal Research Institute WSL, Birmensdorf, Switzerland; 4 Key Laboratory of Geographical Processes and Ecological Security in Changbai Mountains, Ministry of Education, School of Geographical Sciences, Northeast Normal University, Changchun, 130024 China

**Keywords:** Microbial ecology, Wetlands ecology

## Abstract

The bacterial, acidobacterial, and fungal communities in wetlands can undergo perturbations by various human activities, such as disturbances caused by cultivation and during the process of system restoration. In this study, we investigated the relationships between the composition of the soil bacterial, acidobacterial, and fungal communities and the transformation of wetlands by human activities in the Sanjiang Plain. Soil microbial communities were assessed in wetland soils collected from pristine marsh, neighboring cropland (wetland turned into arable land), and land that had been reforested with *Larix gmelinii*. The alpha-diversities of bacteria, Acidobacteria, and fungi were affected by land-use change and were highest in the arable land and lowest in the wetland soils. The soil microbial community structures were also altered with changing land-use. Canonical correlation analyses showed that beta-diversity was significantly affected by soil pH, available phosphorus, soil nitrogen, and total organic carbon. Overall, our results showed that the agricultural cultivation of wetlands changes the available soil carbon, nitrogen, and phosphorus pools, thereby influencing the bacterial, acidobacterial, and fungal diversity and community structure. Once the soil microbial community has been altered by human activity, it might be difficult to restore it to its original state. These findings highlight the importance of effectively maintaining the diversity of soil bacterial, Acidobacterial, and fungal communities despite land use change in order to sustain a microbial community diversity and ecosystem function.

## Introduction

Globally, marsh wetlands are currently threatened by human activities such as land reclamation and forestation^1–3^. These activities can affect water cycle patterns, with downstream effects on deposition and the removal of organic and inorganic matter that result in a reduction of soil organic matter, which can ultimately produce barren areas with low groundwater tables. In recent years, the impacts that cultivation^4^, pollution^5^, and wetland soil microbial community dynamics have on wetland carbon and nitrogen cycles (e.g., greenhouse gas fluxes to the atmosphere)^6^ have attracted increasing attention. It has been demonstrated that soil microbes are crucial players in ecosystem functioning^7^, but there are many microbial taxa in soils. Different microbial groups can differentially impact soil organic matter decomposition, carbon and nitrogen cycling, and the overall stability of the ecosystem^8^. Microbial community structures, with their direct effects on soil functions, reflect changes in the soil nutrient status, and to some extent they are sentinels of the impact of environmental changes^9^. Therefore, understanding the taxonomical and functional dynamics of the soil microbiome will improve our knowledge of the processes of organic matter decomposition, nitrogen fixation, plant–soil interactions, and degradation of organic pollutants^10–12^.

The Sanjiang Plain is the largest wetland in China. It is one of the key regions for global wetland and biodiversity conservation, and the region is also an important grain production and reserve base in China. In recent years, human activities such as cultivation and over-utilization have caused a dramatic decline in the wetland area and have weakened the ecological functions of the remaining wetland. In addition, changes in the soil microbial community structure have been observed^13–15^. The black soil of the Sanjiang marsh wetland in northeastern China has a high humus content and unusual microbial community structures^2^ due to very cold climatic conditions in the winter. Acidobacteria is a new phylum of bacteria that play an important role in soil ecological processes; this phylum accounts for 20–50% of all bacteria, making it one of the most widely distributed and diverse bacterial phyla in natural environments^16–19^. In a previous study, we determined that Acidobacteria was the most abundant bacterial phylum in the soils of the Sanjiang Plain^11^. Therefore, studying the effects of land use on wetland soil microbial communities is very important for better understanding the future changes in wetland ecosystem function and services under global environmental change. Such knowledge will help us to create better policies to effectively protect the Sanjiang wetland.

There is some research on the effects of land- use change on soil physicochemical and biogeochemical characteristics in wetlands and other ecosystems^20–22^. Land-use change can have significant and long-lasting effects on soil carbon and nutrient content, soil texture, and soil pH and that these effects largely arise from changes in the above-ground vegetation and associated management practices across land-use types^20–22^. However, less is known about how wetland change may affect the soil microbiome because of the multiple soil physicochemical factors that are influenced by land-use change. Specifically, many soil variables, such as soil organic matter, texture, salinity and pH, have a significant impact on the soil microbiome, and all of these soil factors may be altered by changes in land-use type^23–26^. Some studies have found that the microbial structure of pristine soil microbes are similar after land use patterns change, indicating that land use patterns do not affect the structure and function of soil microbes^27,28^. However, some studies have found that once land use patterns change, microbial communities cannot recover their natural state even after years of recovery^29^. Peralta *et al*. have shown that wetland soil microbes can only recover from microbial functions in wetland soils only the microbe overcomes the limitation of soil and physicochemical factors^30^. Xu *et al*. found that the soil microbial community structure could not be restored to the original state after the change of land use pattern, but the microbial function was restored indicating functional microbial redundancy^31^. Similarly, our previous study showed that bacterial community metabolic functions changed when wetlands were changed into arable land in the Sanjiang Plain^15^. Overall, the few and sometimes contradictionary studies on the changes in microbial community structure and their drivers (e.g. soil physicochemical properties) limit our understanding of soil biogeochemical processes after the reclamation of marshes in farmland and plantation.

To gain deeper insight into how soil biogeochemical processes are affected by wetland changes, we used a typical degraded wetland in Sanjiang plain, northeastern China and selected two dominant typical habitats by human activity disturbance: a restored forest (planted on degraded wetlands in 2000) and a managed arable land (soybean planted in 2007), which is a typical example of an inland freshwater marsh that is dramatically changing. Using Illumina MiSeq sequencing technology we examined changes in soil bacterial, acidobacterial and fungal communities, and their relationships with soil physiochemical properties in three different land use types originating from marsh wetland cultivation. Our specific objectives were (i) to elucidate the changes in soil microbial communities in marshes that have experienced long periods of cultivation and forest plantation, (ii) to investigate the relationship between microbial communities and soil properties, and (iii) to gain insight into soil biogeochemical processes after marsh changes. The data presented here will help quantify the influence of land-use pattern changes on wetland soil microbes in northeastern China. This research is of great practical significance for improving environment management and utilization of microbial resources, and provides scientific guidance for wetland protection and management in northeastern China.

## Results

### Soil bacterial, Acidobacterial, and fungal community composition

**Figure 1:**
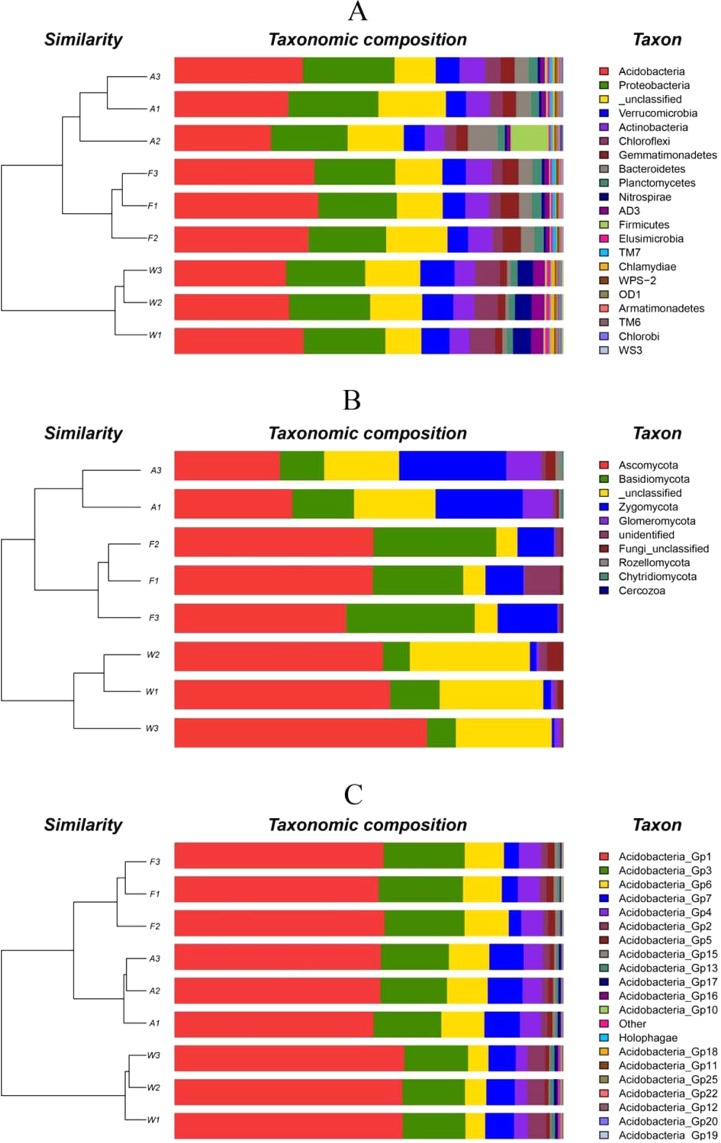
Histogram and cluster analysis of soil bacterial (panel A), fungal (**B**) and acidobacterial (**C**) communities in different land use types, for three forests samples (F1–F3), three wetland samples (W1–W3) and three arable land samples (A1–A3).

A total of 3917 bacterial OTUs included here belonged to 40 taxonomic groups. In decreasing order of abundance, the six most abundant phyla were as follows: Acidobacteria (31.8%) > Proteobacteria (21.1%) > Verrucomicrobia (6.4) > Actinobacteria (4.1%) > Chlorobi (3.2%) > Gemmatimonadetes (3.2%) (Fig. 1A, Table 1). These were considered to constitute the main bacterial phyla and, together with a significant fraction of unclassified OTUs (9%), they represented approximately 80% of the data from bacterial phyla. There were 30 phyla with a relative abundance <1%. The similarity of soil bacterial community structure was divided into two parts (Fig. 1A): wetlands (W1–W3) had similar bacterial community structures, while arable lands (A1–A3) and forests (F1–F3) had similar bacterial community structures.

**Table 1:** Differences in relative abundances of all detected phyla in the different land use habitat types in the Sanjiang Wetland, northeastern China.

Taxonomy		Relative abundance (mean ± sd; n = 3)			One-way ANOVA
Domain	Phylum	Wetland	Forest	Arable land	
Bacteria	Acidobacteria	30.5 ± 1.38	35.8 ± 0.74	29.0 ± 2.36	0.058 ns
Bacteria	Proteobacteria	20.7 ± 0.21	20.3 ± 0.24	22.2 ± 1.24	0.259 ns
Bacteria	unclassified	12.3 ± 1.53	13.2 ± 1.27	14.1 ± 1.96	0.732 ns
Bacteria	Verrucomicrobia	8.0 ± 0.4	5.7 ± 0.22	5.5 ± 0.30	0.003**
Bacteria	Actinobacteria	5.3 ± 0.09	6.4 ± 0.15	6.0 ± 0.42	0.063 ns
Bacteria	Chloroflexi	6.3 ± 0.09	2.7 ± 0.15	3.4 ± 0.424	0.000**
Bacteria	Gemmatimonadetes	1.9 ± 0.01	4.5 ± 0.21	3.3 ± 0.17	0.000**
Bacteria	Bacteroidetes	1.0 ± 0.06	3.5 ± 0.11	5.0 ± 1.31	0.027*
Bacteria	Planctomycetes	1.6 ± 0.06	2.4 ± 0.04	2.1 ± 0.14	0.002**
Bacteria	Nitrospirae	4.3 ± 0.17	0.6 ± 0.08	0.6 ± 0.04	0.000**
Bacteria	AD3	3.2 ± 0.10	1.2 ± 0.08	1.1 ± 0.07	0.000**
Bacteria	Firmicutes	0.5 ± 0.00	0.3 ± 0.03	3.6 ± 3.07	0.389
Bacteria	Chlamydiae	1.0 ± 0.03	0.2 ± 0.01	0.5 ± 0.02	0.000**
Bacteria	Others	3.5 ± 0.08	3.1 ± 0.08	3.5 ± 0.08	0.018*
Fungi	Ascomycota	57.99 ± 6.06	48.74 ± 3.98	28.71 ± 2.26	0.217 ns
Fungi	Zygomycota	1.41 ± 0.68	11.48 ± 3.33	24.96 ± 3.63	0.032*
Fungi	unclassified	27.40 ± 3.15	5.69 ± 0.23	20.12 ± 1.22	0.078 ns
Fungi	Basidiomycota	9.01 ± 3.21	29.31 ± 5.27	13.62 ± 3.16	0.337 ns
Fungi	Glomeromycota	1.11 ± 0.38	0.32 ± 0.04	8.36 ± 0.82	0.007**
Fungi	unidentified	1.08 ± 0.84	3.75 ± 4.66	0.98 ± 0.24	0.024*
Fungi	Others	0.03 ± 0.01	0.19 ± 0.06	0.55 ± 0.25	0**
Fungi	Fungi_unclassified	1.98 ± 1.97	0.51 ± 0.08	1.60 ± 1.31	0.067
Fungi	Rozellomycota	0.00 ± 0.00	0.02 ± 0.01	1.10 ± 0.88	0.000**
Acidobacteria	Acidobacteria_Gp1	28.95 ± 0.24	28.62 ± 0.95	31.08 ± 0.22	0.035*
Acidobacteria	Acidobacteria_Gp10	0.03 ± 0.00	0.10 ± 0.01	0.20 ± 0.05	0.015*
Acidobacteria	Acidobacteria_Gp11	0.08 ± 0.06	0.01 ± 0.02	0.00 ± 0.00	0.162 ns
Acidobacteria	Acidobacteria_Gp12	0.02 ± 0.00	0.01 ± 0.00	0.00 ± 0.00	0.084 ns
Acidobacteria	Acidobacteria_Gp13	0.49 ± 0.00	0.30 ± 0.02	0.28 ± 0.05	0.040*
Acidobacteria	Acidobacteria_Gp15	0.18 ± 0.01	0.43 ± 0.04	0.60 ± 0.06	0.084 ns
Acidobacteria	Acidobacteria_Gp16	0.13 ± 0.01	0.13 ± 0.01	0.09 ± 0.00	0.400 ns
Acidobacteria	Acidobacteria_Gp17	0.36 ± 0.05	0.30 ± 0.03	0.12 ± 0.00	0.092 ns
Acidobacteria	Acidobacteria_Gp18	0.10 ± 0.01	0.00 ± 0.00	0.00 ± 0.00	0.053 ns
Acidobacteria	Acidobacteria_Gp19	0.02 ± 0.01	0.00 ± 0.00	0.00 ± 0.00	0.018*
Acidobacteria	Acidobacteria_Gp2	2.26 ± 0.08	0.92 ± 0.05	0.91 ± 0.12	0.244 ns
Acidobacteria	Acidobacteria_Gp20	0.01 ± 0.00	0.01 ± 0.00	0.00 ± 0.00	0.521 ns
Acidobacteria	Acidobacteria_Gp22	0.02 ± 0.00	0.00 ± 0.00	0.00 ± 0.00	0.224 ns
Acidobacteria	Acidobacteria_Gp23	0.00 ± 0.00	0.00 ± 0.00	0.00 ± 0.00	ns
Acidobacteria	Acidobacteria_Gp25	0.05 ± 0.01	0.03 ± 0.00	0.01 ± 0.00	0.071 ns
Acidobacteria	Acidobacteria_Gp3	7.97 ± 0.07	9.47 ± 0.09	12.21 ± 0.44	0.018*
Acidobacteria	Acidobacteria_Gp4	1.61 ± 0.15	2.81 ± 0.11	3.31 ± 0.04	0.200 ns
Acidobacteria	Acidobacteria_Gp5	0.47 ± 0.03	0.65 ± 0.05	1.03 ± 0.02	0.168 ns
Acidobacteria	Acidobacteria_Gp6	2.60 ± 0.09	5.81 ± 0.14	6.11 ± 0.42	0.031*
Acidobacteria	Acidobacteria_Gp7	3.56 ± 0.14	4.88 ± 0.08	2.15 ± 0.28	0.200 ns
Acidobacteria	Holophagae	0.11 ± 0.01	0.07 ± 0.01	0.01 ± 0.00	0.272 ns
Acidobacteria	Other	0.22 ± 0.02	0.06 ± 0.01	0.05 ± 0.01	0.112 ns

Note: The level of significance determined by one way ANOVA is listed (**P < 0.01, ^*^P < 0.05, ns: not significant).

A total of 1818 fungal OTUs included here belonged to 9 phyla groups. In decreasing order of abundance, the four most abundant fungal phyla were Ascomycota (47.2%) > Basidiomycota (17.8%) > Zygomycota (11.1%) > Glomeromycota (2.6%). These were considered to constitute the main fungal phyla and, together with a significant fraction of unclassified OTUs (17.4%), they represented approximately 90% of the data from fungal phyla. There were three phyla with a relative abundance < 1% (Fig. 1B, Table 1). Meanwhile, there was a high abundance of unclassified phyla in the wetland soils (Fig. 1B, Table 1). The Zygomycota was the main phylum in arable land soils, while Basidiomycota was most abundant in forest soils (Fig. 1B, Table 1). The similarity of these three land use types in soil fungal community structure was consistent with that of bacteria (Fig. 1B). The fungal community structures of wetland soils (W1 to W3) were different from those of arable land (A1 to A3) and forest (F1 to F3), which had similar fungal community structures.

A total of 1120 Acidobacterial OTUs included here belonged to 25 subgroups. In decreasing order of abundance, the six most abundant phyla were: Acidobacteria_Gp1 (29.6%) > Acidobacteria_Gp3 (9.9%) > Acidobacteria_Gp6 (4.8%) > Acidobacteria_Gp7 (3.5%) > Acidobacteria_Gp4 (2.6%) > Acidobacteria_Gp2 (1.4%) (Fig. 1C, Table 1). These phyla were considered to constitute the main Acidobacterial community and, together with a significant fraction of unclassified OTUs (0.1%), they represented approximately 51.9% of the data from total sequences. In contrast, 49.1% of the total sequences were not of an Acidobacterial origin and were removed prior to subsequent analysis. There were 15 phyla with a relative abundance <1%. Acidobacteria_Gp13, Acidobacteria_Gp18, Acidobacteria_Gp19, and Acidobacteria_G22 were more abundant in wetlands compared with arable lands and forests. Meanwhile, Acidobacteria_Gp6, Acidobacteria_Gp15, and Holophagae were low abundance in the wetlands compared with arable lands and forests (Fig. 1C, Table 1). The soil Acidobacterial community structure similarity of the three land-use types was consistent with those of bacteria and fungi. Acidobacterial community structures in wetlands were different from those in arable lands and forests, which had similar Acidobacterial community structures (Fig. 1C).

### Alpha-diversity

The alpha-diversities of the three different land-use patterns are shown in Table 2. For bacteria, the Shannon-Wiener, Chao, and Simpson’s indices differed significantly (P < 0.01) between wetlands, forests and arable lands (Table 2). For Acidobacteria, the Shannon-Wiener and Chao indices were significantly different (P < 0.01) between wetlands and the other two land use patterns. For fungi, the Shannon-Wiener index and the Simpson’s index did not significantly differ (P > 0.05) between the wetlands, forests, and arable lands (Table 2). However, the Chao index differed significantly among the three different land use patterns (P < 0.01) (Table 2).

**Table 2:** The α-diversity indices (mean ± sd; n = 3) of bacterial, acidobacterial and fungal OTUs obtained from the soils of three different land use types in the Sanjiang Wetland, northeastern China.

Organisms	Type	Chao	Shannon	Simpson
Bacteria	Wetland	2894.4 ± 13.8^b^	5.9 ± 0.02^c^	0.0078 ± 0.00012^a^
	Forest	2748.2 ± 29.0^c^	6.1 ± 0.03^b^	0.0063 ± 0.00012^b^
	Arable land	3187.8 ± 55.7^a^	6.3 ± 0.02^a^	0.0054 ± 0.00018^c^
Acidobacteria	Wetland	916.5 ± 8.3^b^	4.9 ± 0.00^a^	0.0177 ± 0.0009^a^
	Forest	848.7 ± 9.2^a^	5.2 ± 0.03^b^	0.0203 ± 0.0011^b^
	Arable land	925.0 ± 5.7^b^	5.2 ± 0.02^b^	0.0189 ± 0.0005^ab^
Fungi	Wetland	405.2 ± 74.3^b^	3.3 ± 0.46^a^	0.0970 ± 0.0361^a^
	Forest	666.5 ± 37.5^a^	3.9 ± 0.12^a^	0.0447 ± 0.0051^a^
	Arable land	696.5 ± 110.5^a^	4.2 ± 0.29^a^	0.0650 ± 0.0207^a^

Note: Different letters denote significant differences between habitats at P < 0.05. α-diversity indexes were calculated at the OTU level.

### Beta-diversity

**Figure 2:**
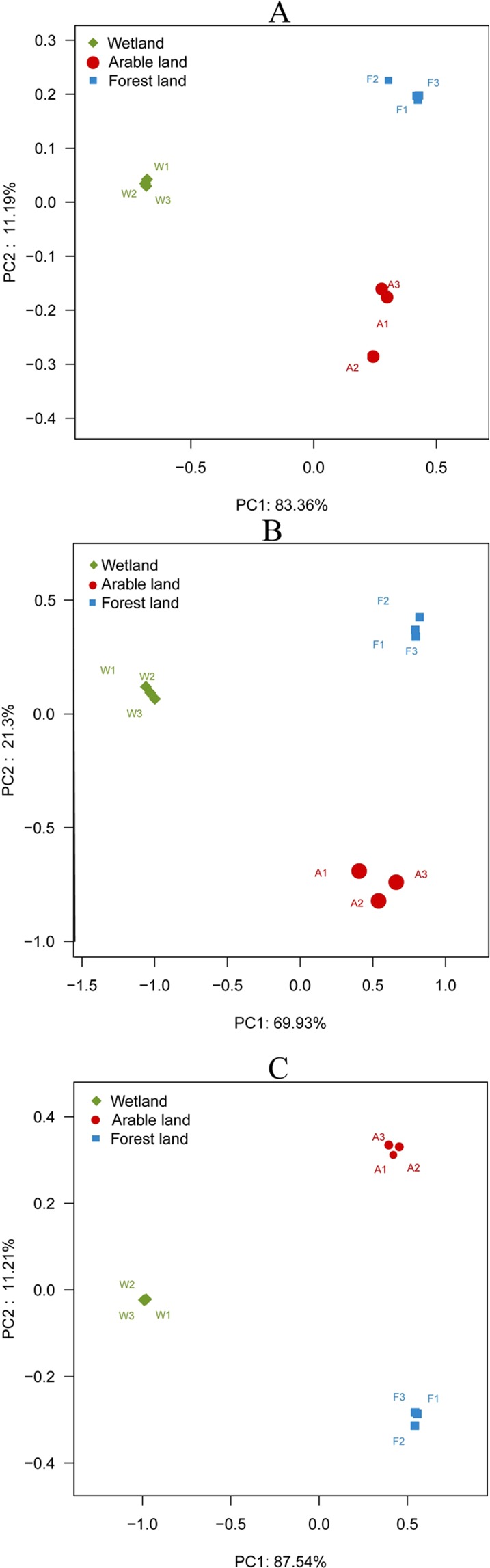
PCoA diagram of bacterial (**A**), fungal (**B**) and acidobacterial (**C**) communities identified from soils in wetland (green), forest (blue), and arable land (red).

In the analysis of beta-diversity, wetlands were separated from the other two land-use types, suggesting a clear distinction in the bacterial community structures between wetlands and forests and arable land (Fig. 2A). This finding was supported by the results of a principal coordinates analysis (PCoA) and a PERMANOVA (Fig. 2A and Table 3). In general, the PCoA explained 90.71% of the variation in the bacterial communities.

**Table 3:** PERMANOVA analysis of bacteria, acidobacteria and fungi between three different land use types in Sanjiang plain, northeast of China.

	F	R^2^	p
Bacteria	25.6**	0.90	0.003
Acidobacteria	89.1**	0.97	0.004
Fungi	12.2	0.80	0.080

The PCoA revealed the beta-diversity of the Acidobacterial communities in the wetland, forest, and arable soils (Fig. 2B and Table 3). Overall, the PCoA explained 96.80% of the variation for the Acidobacterial communities. Wetland samples were clustered together and were well separated from the samples from the other habitats (forest and arable land). These results suggest that land use change significantly impacted the soil Acidobacterial communities. Fungal community structures in the wetland samples were also clearly different from the arable land and forest samples (Fig. 2C and Table 3). Overall, the two PCoAs explained 85.83% of the variation in the fungal communities.

### Bacterial, Acidobacterial, and fungal beta-diversity in relation to soil physical and chemical properties

**Figure 3:**
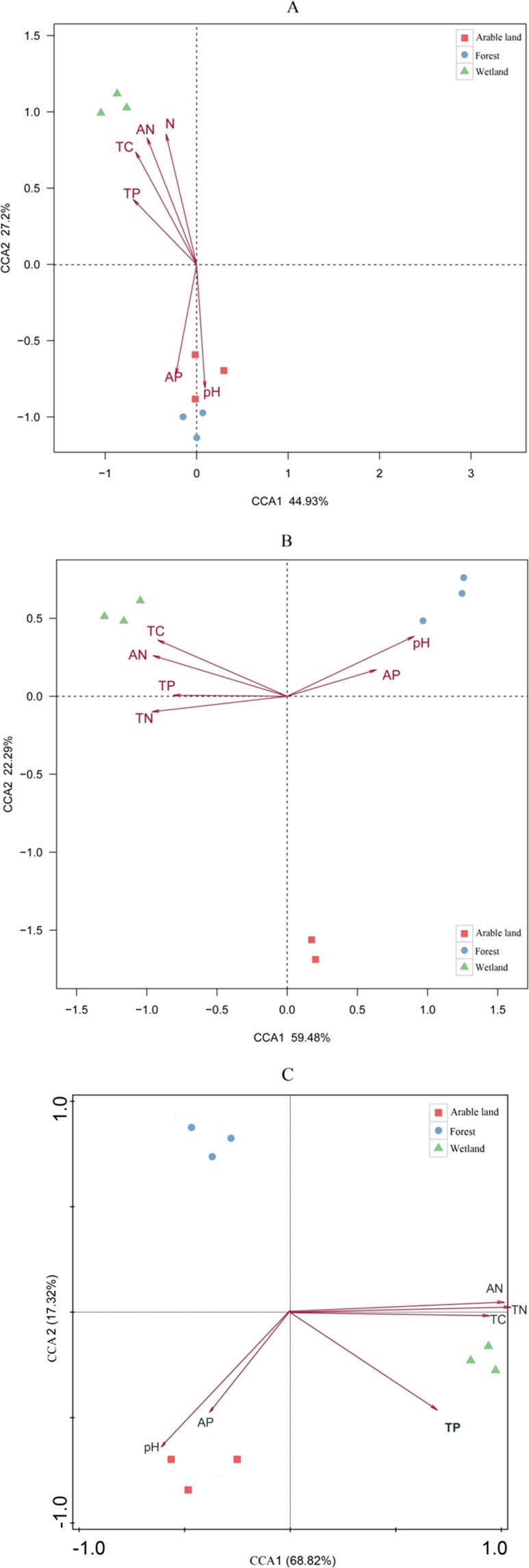
CCA of soil bacterial (**A**), fungal (**B**) and acidobacterial (**C**) community and environmental factors. Samples from wetland (W1–W3), forest (F1–F3) and arable land (A1–A3) are shown. TC: total organic carbon; TN: total nitrogen; AN: available nitrogen; AP: available phosphorus; TP: total phosphorus.

Canonical correlation analysis (CCA) of the bacterial data revealed that soil physicochemical properties explained most of the variance in the CCA, for example, the first two axes explained 72.13% (Fig. 3A). The forest and arable land bacterial communities were positively correlated with soil available phosphorus (AP) and soil pH, while the wetlands were positively correlated with total phosphorus (TP), total carbon (TC) total nitrogen (TN), and available nitrogen (AN).

CCA of the fungal data showed that the first two axes explained 81.77% of the total variance (Fig. 3B). The forest and arable land were positively correlated with available phosphorus (AP) and the soil pH, while wetlands were positively correlated with TP, TC, AN, and TN.

CCA of the Acidobacterial data showed that the first two axes explained 86.14% of the variance (Fig. 3C). The wetlands were positively correlated with TC, TN, TP, and AN, while arable land and forest were positively correlated with soil pH and AP.

## Discussion

### Bacterial diversity, community composition, and the soil environment

In this study, we used Illumina MiSeq sequencing technology to determine the soil microbial community composition in three different land use types. In total, we resolved 40 phyla from soils collected from the Sanjiang Plain, with noticeable differences between wetland, arable land and forest soils. There were significant differences in the microbial communities between the different land-use types (Fig. 1A). By cluster analysis, we established that the communities thriving in arable land soils were relatively similar to those in forest soils—despite the obviously different vegetation in these land use types—indicating that vegetation type is not a good indicator of soil bacterial community structures. The observed bacterial community composition of the wetlands was markedly different from that of the other two soil types. This suggests that changing wetlands into forests or arable land altered the soil properties considerably, with changes in the soil microbial population structure occurring as a consequence^32^. The variation in bacterial composition indicated that the land-use change disturbed the ecological stability of the original (wetland) microbial community and changed the habitat conditions, which resulted in shifts in the dominant microbial phyla and community diversity^33^.

We found that major microbial phyla were overrepresented in wetland soils, with a high abundance relative to that in the other land-use types (Fig. 1A). For example, there were significantly higher relative abundances of Chloroflexi and Chlamydiae in the wetland soils compared to the forest and arable land soils. In contrast, the relative abundance of Bacteroidetes and Tenericutes was significantly higher in forest soils, and Planctomycetes and Gemmatimonadetes were higher in arable land soils (Table 1). Our findings are consistent with the literature, indicating that different habitats contain specific bacterial taxa^34,35^. Wetland soils are characterized by high soil moisture and low pH; this habitat may be suitable for some bacterial phyla such as Chloroflexi and Chlamydiae. For example, Zhang studied the rhizosphere bacterial diversity of three *Phragmites australis* ecotypes in the Hexi Corridor, China, and found that members of the Chlamydiae phylum only existed in the swamp reed wetland^36^. Han investigated the microbial community composition in Ebinur Lake, China, and found that Chlamydiae were the main phylum correlating with soil moisture and pH^37^. In our research, the soil moisture of the wetland was significant higher (p < 0.05) than that of the arable land and forest soils, and the high soil moisture might be a habitat more suitable for Chlamydiae. Hence, the abundance of Chlamydiae in the wetland soils was higher than in the other habitats. The soil microbial community structures changed from wetlands to arable land and forest because of the changes of soil hydrothermal and nutrient conditions^38^. Changes in the soil microbial community structure are a direct manifestation of the adaptation of that microbiota to changed conditions (due to human activities) that accelerate the decomposition of soil organic carbon and promote the utilization of soil nutrients by plants^39^.

Diversity indices quantify the diversity of soil microbial communities. Our results indicated that the Shannon-Wiener index of bacteria from the wetland soil was lower than from the other two soil types, suggesting that land-use changes from wetland to forest or arable land will increase the soil bacterial diversity. This result is consistent with Yu *et al*., who found that soil bacterial diversity was higher in agricultural land (Shannon-Wiener index 3.49–3.69) than in natural restoration land (3.34–3.44)^40^. The latitude of the area they analyzed is similar to that of the Sanjiang Plain, and the soil conditions are also similar, so the results of their study are comparable to ours. Xu *et al*. also reported that cultivated land had higher bacterial diversity than pristine marshland on the Sanjiang Plain^41^.

Moreover, Hartman *et al*. found that the diversity of the soil bacterial community was lower in natural wetlands than in arable land on the North Carolina coastal plain, finding that fertilization practices increased the soil pH, which resulted in an increase in the bacterial diversity in arable land^42^. There are many studies that report soil pH is highly positively correlated with soil bacterial diversity^43,44^. However, Ausec *et al*. found that the soil bacterial diversity was higher in bog soils with low pH values but high organic carbon content, and they concluded that the soil organic carbon was the key factor determining the soil bacterial diversity^45^. The importance of soil organic carbon^45^ is not supported by the present study, because the soil pH and total organic carbon content were 5.8 and 25.6 for the arable land (high diversity), and 5.5 and 52.7 for the wetland (low diversity), respectively (Table 4). Our study suggests that soil pH rather than soil organic carbon content plays a more important role in affecting the soil bacterial diversity.

**Table 4:** Physicochemical properties of soils from three different land use habitat types in the Sanjiang Wetland, northeastern China.

Land use type	pH	Total organic carbon (g/kg)	Total nitrogen (g/kg)	Available nitrogen (mg/kg)	Total phosphorus (mg/kg)	Available phosphorus (mg/kg)	Soil gravimetric moisture (%)
Wetland	5.5 ± 0.0^c^	52.7 ± 1.3^a^	4.3 ± 0.8^a^	455.3 ± 29.6^a^	6.4 ± 1.2^a^	26.3 ± 1.8^a^	52.8 ± 1.0^a^
Arable land	5.8 ± 0.1^b^	25.6 ± 1.4^b^	3.1 ± 0.6^b^	197.6 ± 7.5^b^	3.7 ± 0.7^b^	26.7 ± 3.6^a^	32.9 ± 3.1^c^
Forest	7.4 ± 0.1^a^	25.1 ± 1.5^b^	1.8 ± 0.3^c^	143.8 ± 7.2^c^	3.2 ± 0.5^b^	32.7 ± 4.5^a^	47.0 ± 1.2^b^

Note: Statistically significant differences (P < 0.05; *n* = 3) between habitats is indicated by different superscript letters a–c in the same column.

In our study, changes in land use patterns resulted in changes in soil physicochemical properties (P < 0.05) (Table 1). Soil samples from the wetlands had a higher mean moisture content and a lower mean pH than those from the arable land or forest (Table 1). Soil bacterial diversity was affected by soil pH and soil moisture conditions in our study. This result seems counterintuitive because the concentrations of TC, TN, AN, and TP decreased significantly from wetland to forest. This phenomenon may be due to perennial accumulation of organic matter in the swamp meadow wetland soils. Thus, the soil bacteria cannot use the nutrient deposits despite their high abundance in the wetlands. Under new land type regimes, however, the original form of these soil nutrients changed, which provided favorable (more oxic) conditions for the utilization of C and N by soil microbes. The limiting factor of soil microbial diversity in the wetland soils is anaerobic conditions rather than the C and N content.

### Acidobacterial diversity, community composition, and the soil environment

We found that the Shannon-Wiener diversity of Acidobacteria from the wetland soils was lower than that from the soils of the other two habitats. This was consistent with the pattern found for total bacteria, demonstrating that land-use change can lead to variations in bacterial and Acidobacterial α-diversity. However, this tendency in total bacterial α-diversity was different with Acibobacterial α-diversity. That is, the Shannon-Wiener diversity of the total bacterial community in wetland soil was significantly different from that in the forest and arable land (Table 2). For Acidobacteria, however, the Shannon-Wiener index for the wetland soils was significantly different from that of the arable land, but not from that of the forest. Our study indicated that soil AP content and pH were the major soil parameters driving bacterial diversity in the arable and forest soils, while TP, TC, TN, and AN were the key soil parameters determining soil Acidobacterial diversity in the wetland soils. Our data suggest that the soil Acidobacterial community was strongly affected by soil pH^46,47^. Similar to our findings, land use change altered the Acidobacterial community diversity in soybean cropland and adjacent native forests in Amazon forest soils^48^. Naether *et al*. analyzed the Acidobacterial community diversity of three forest and grassland areas in Germany; they found significant differences between the grassland and forest, including significant differences between different sites within the same environment^49^.

In our study, Gp1, Gp3, Gp6, Gp7, Gp4, Gp2, Gp5, and Gp15 (listed in decreasing order from highest to lowest relative abundances >1%) were the main Acidobacterial subgroups in the three land use types investigated. The composition of Acidobacteria in our soil samples was comparable to the relative abundances of dominant Acidobacterial subgroups found in other studies of Acidobacterial communities^19,47,49^. Generally, Gp1, Gp2, Gp3, Gp4, Gp6, and Gp16 have been found to be the most abundant subgroups in most soils studied, but the relative abundances of those subgroups varied among soil samples^50,51^. Our study was nearly consistent with these previous results. A notable distinction, however, is that Gp2 has been frequently detected in forest soils in previous studies, accounting for 11.5% of Acidobacteria in Xishuangbanna forest soils^52^, 29.0% in Changbai mountain soils^53^, 5.5–33.2% in Shennongjia forest soils^54^, and it was a main dominant subgroup in *Pinus sylvestris* forests^55^. However, in this study, the forest and arable land soils had less Gp2, but the relative abundance in these soils was only approximately 0.98% and 0.91% (Table 2). Eichorst^56^ *et al*. also found that there were rarely GP2 or many other subgroups (GP1, GP3, GP4, and GP6) in agricultural soils. Navarrete^19^ *et al*. found that the relative abundance of GP2 was higher in Amazon forest soils than agricultural soils, implying that agricultural practices could decrease the soil GP2 abundance. We need to examine the effects of agronomic actives on soil Acidobacterial subgroup GP2 in future studies.

In our study, the community composition of Acidobacteria was affected by the pH, AP, AN, TP, and TC in the soil. Further, the relative abundances of GP1, GP3, GP4, GP5, GP6, and GP15 were positively correlated with soil pH, while the abundance of GP2 and GP7 were negatively correlated with pH (Fig. 3B). Our results are similar with the results of Jones^46^ and Liu^47^. In addition, Liu^47^ *et al*. demonstrated that soil pH was a key soil physicochemical property that affected soil Acidobacterial subgroup relative abundance. The soil pH adjusted the abundance of Acidobacteria subgroups in the black soils of northeast China. Other than soil pH, previous studies have found that soil environmental factors had a strong relationship with the abundance of soil Acidobacterial subgroups^19,56^. For example, Navarrete^19^ found that high aluminum content could limit the relative abundance of the GP6 and GP7 subgroups of Acidobacteria, but high contents of soil Ca, Mg, Mn, and B promoted the relative abundance in the Amazon forest. Eichorst^56^ *et al*. found that the relative abundance of Gp 4 was significantly negatively correlated with soil carbon concentration in agricultural and managed grassland soils. Like these studies, our study observed that soil Acidobacterial subgroups showed significant correlations with soil physicochemical properties, with the exception of soil pH. This indicates that soil physicochemical properties, such as pH, TC, TN, TP, and AP could affect the community composition of Acidobacterial communities.

### Fungal diversity, community composition, and the soil environment

Fungi play important roles in soil ecosystems, and the community composition of soil fungi is affected by management^57^ and soil nutrient status^58^. In this study, we observed that fungal diversity was higher in arable lands and forests than in the wetlands, which is consistent with previous research^59^. The high soil water content of wetlands results in decreased soil oxygen content. These conditions most likely restrain the survival of fungi, resulting in decreased soil fungal diversity.

The diversity of soil fungi in arable lands and forests was significantly higher than that in the wetlands, presumably because the condition of the soil environment in the arable land was influenced by disturbances such as agricultural tillage, which resulted in a higher soil oxygen content than the wetland soils. Moreover, because of the abundance of litter inputs and the higher soil oxygen content, the diversity of soil fungi in the forest was also higher than that in the wetland^60^. The high plant diversity in the forest may have also increased the soil fungal biomass and diversity^38^.

Our results showed that the fungal phyla structures were affected by various soil physicochemical properties in the three studied land-use patterns of the Sanjiang Plain. A CCA showed that soil pH and SOC, TP, AN, and TN had obvious effects on the fungal community structure in the different land-use types (Fig. 3C). The variation in the fungal composition among land use types indicated that soil fungal structure changed when the original wetland was converted into arable land and forest. Land use change resulted in a change in the aboveground vegetation, which caused new habitat conditions and a concomitant increase or decrease of specific fungal taxa (e.g., root-associated), which, in turn, changed the fungal community composition^61^. Soil organic C and total N are important carbon and nitrogen sources for fungi. Forests and arable lands are rich in plant litter that can promote fungal growth^62^. In addition, some studies have indicated that fungi might promote their growth by improving their water use efficiency in acidic environments^63^. Soil P is energy for soil fungal communities, but it is also an important resource for plant growth^64^. In our study area, shifts in land use changed the soil physiochemical factors—such as pH, TC, TN, and TP—which, in turn, affected the soil fungal community composition.

Vegetation was found to affect the soil microbial community structure^13,65^. Underground ecosystems with the roots of various plants may have a marked effect on soil microbial structures^61^. Therefore, the relationships of soil microorganisms, aboveground vegetation, and the soil environment need to be investigated to clarify the interactions between and among them in order to better understand the ecosystem effects on soil microorganisms. Our results showed that there were significant differences in soil fungal structure among the three land use types. This may, at least partly, indicate an important effect of vegetation type associated with land use change on the structure and function of the soil fungal community. Although we widely tested the effects of seven physicochemical properties on soil bacterial, Acidobacterial, and fungal diversity in the present study, other abiotic factors such as soil temperature and soil oxygen content might also be important, and thus future studies should quantify their impacts on soil microorganisms.

Many studies proved that different land use patterns changed vegetation composition, gas permeability, water content, and microclimate characteristics of soil, and these change would affect the microbial community structure and diversity and function^66–68^. Changes in land use patterns have changed the original niche of soil microbes, therefore, resulting in corresponding changes in the soil microbial community structure. However, these conclusions were only obtained through some statistical tests, and the real situation cannot be unified because of its complexity^69^. For example, although our study found that soil organic carbon significantly affected soil microbial structure, but the effects of organic carbon interacted with other factors, and other environmental factors also had a significant impact on soil microbes. Therefore, how to distinguish and analyze the microbial community changes that are affected by each single soil factors is very difficult.

We concluded that soil microbial communities were affected by soil physicochemical properties, but the content of soil physicochemical properties did not affect the soil microbial communities and the quality and type of soil physicochemical properties mainly affected the soil microbial communities^70,71^. In addition, the identity of these soil parameters in the original ecosystem depends on the original nature of each ecosystem in different areas.

## Conclusion

We observed differences in the soil properties as well as the characteristics of the soil bacterial, Acidobacterial and fungal communities among the three different land use types in Sanjiang plain. The Shannon diversity index of both soil bacteria and Acidobacteria varied significantly among the three land use types. The bacterial, Acidobacterial and fungal community structures showed similar distinctions between arable land and forest land, but differed with marsh wetland. Our data suggest that soil pH and resource availability (soil organic carbon, total nitrogen, available nitrogen, and total phosphorus concentrations) are the primary drivers of soil bacterial and Acidobacterial and fungal community compositions along the land use changed. This study improved our understanding of soil microbial structure in a typical wetland ecosystem, and the results can be used to predict the impact of land use change from wetland to farmland and forest plantation on the soil microbiome.

## Materials and Methods

### Site description

The study was performed in the Sanjiang Plain (47°35′N, 133°31′E), northeastern China. The local average monthly temperature ranged from −21.6 °C in January to 21.5 °C in July, with an annual mean temperature of 1.9 °C. The mean annual precipitation was approximately 560 mm, of which 80% occurred from May to October. We selected three land use types (habitats) for this study: wetland, arable land developed from wetlands, and a planted forest on degraded wetlands.

The wetland habitat was a pristine marsh meadow wetland that was seasonally flooded from May to August, and was the typical wetland type occurring in the Sanjiang Plain where *Deyeuxia angustifolia* is the dominant plant species^13^. The arable land habitat was converted from a pristine marsh meadow wetland into a soybean plantation in 2007. The soybean plantation was fertilized with 370 kg ha^−1^ y^−1^ of fertilizer (N:P:K) each year in May. The forest habitat was plantations of *Larix gmelinii* that was planted on degraded wetlands in 2000. At the time of this study in 2016, the average height of the *Larix gmelinii* was about 7 m, the diameter at breast height was about 12 cm, and the average density was 1600 stems ha^−1^. No fertilization or forest management was conducted in this forest plantation. The wetland and arable land habitat types were approximately 400 m apart, and the forest plantation was located between them at a distance of approximately 200 m from each of the other two habitat types. The wetland, forest, and arable land covered a total area of 3000 m^2^, 2000 m^2^, and 1000 m^2^, respectively.

### Soil sampling

Soil samples (0–20 cm depth) were taken on 15 October 2016. Three plots (10 m × 10 m) were established in each habitat, and the distance between any two plots within the same habitat was >50 m. We randomly designated five smaller quadrats (1 × 1 m each) within each standard quadrat. We measured three individual replicates per site, whereas in each site, five individual soil samples were taken and pooled. Soil samples were collected from 5 locations inside each plot using a sterile soil drill and then pooled to obtain a mixed soil specimen (approximately 1 kg of fresh soil) for each plot. We stored field soil samples in an ice box with a temperature near 4 °C, which was transported immediately to the lab for DNA extraction as soon as possible to avoid any freeze-thawing that would affect the integrity of the soil DNA. In the laboratory, approximately 10 g of fresh soil from each sample was transferred into a sterile 50 mL Falcon tube and kept at −80 °C for microbial analysis. The remaining soil was air dried for soil physiochemical analyses.

### Determination of soil physiochemical properties

The pH was measured using a pH meter after mixing the soil with water (1:5 w/v) for 30 min. The total organic carbon (TC) and total nitrogen (TN) concentrations of the soil samples were determined using an elemental analyzer (VarioEL III, Elementar Analyses System, Hanau, Germany). Total phosphorus (TP), available phosphorus (AP), and available nitrogen (AN) were digested with H_2_SO_4_-HClO_4_, 0.5 M NaHCO_3_, and 2.0 M KCl in succession and then assayed using a continuous flow analytical system (SKALAR SAN^++^, the Netherlands). Soil moisture content was measured gravimetrically. The physiochemical properties of the soil samples are summarized in Table 4.

### Soil DNA extraction, PCR amplification, and high-throughput sequencing

DNA was removed from a 0.5 g frozen soil specimen with a MOBIO Power Soil DNA Isolation Kit (Mo Bio Laboratories, Carlsbad, CA, USA) based on the company’s directions. The extracted DNA was diluted in 100 μL TE (10 mM Tris–HCl, 1 mM EDTA, pH 8.0) and then kept at −20 °C prior to use. The DNA was quality checked and quantified using a Nano Drop ND-1000 spectrophotometer (Thermo Fisher Scientific, Waltham, USA). We extracted the DNA of each soil sample individually and performed PCR in triplicate for each DNA sample. We amplified the V3-V4 region of bacterial 16S rRNA^72^ as well as the ITS1 region of fungal ITS rRNA^73^. We selected the primers ACIDO (5′GCTCAGAATSAACGCTGG3′)/342r (5′CTGCTGCSYCCCGTAG3′) to amplify the Acidobacterial specific phyla^74^.

The bacterial PCR reactions were performed in a 25 uL mixture containing 0.5 uL of each primer (30 μmol L^−1^), 1.0–1.5 μL of template DNA (10 ng), and 22.5 μL of Platinum PCR SuperMix (Invitrogen, Shanghai, China). The amplification procedure was as follows: 95 °C for 3 min, followed by 35 cycles at 95 °C for 30 s, 55 °C for 30 s, and 72 °C for 30 s, followed by an extension at 72 °C for 10 min.

The fungal PCR reactions were performed in a 25 μL mixture containing 0.5 μL of each primer (30 μmol L^−1^), 1.5 μL of template DNA (10 ng), and 22.5 μL of Platinum PCR SuperMix (Invitrogen, Shanghai, China). The amplification procedure was as follows: 95 °C for 5 min, followed by 30 cycles at 95 °C for 30 s, 55 °C for 30 s, and 72 °C for 30 s, followed by an extension at 72 °C for 10 min.

The Acidobacterial PCR reactions were performed in a 25 uL mixture reaction system similar to that used for bacteria. The amplification procedure was as follows: 95 °C for 5 min, followed by 35 cycles at 95 °C for 60 s, annealing at 63 °C for 60 s, and extension at 72 °C for 60 s, with a final extension at 72 °C for 5 min.

Each sample was amplified in triplicate, and amplicons were removed from 2% agarose gels, purified with the Axy Prep DNA Gel Extraction Kit (Axygen Biosciences, Union City, CA, USA) based on the company’s directions, and then quantified with QuantiFluor-ST (Promega, Madison, WI, USA). The three purified amplicons from one DNA sample were pooled at equimolar concentrations and paired-end sequenced on a MiSeq PE 300 platform (Illumina, San Diego, CA, USA) based on standard protocols at BIONOVA BioPharm Technology Co., Ltd., Beijing, China.

### Sequence analysis

Bacterial, Acidobacterial and fungal raw fastq files were de-multiplexed, quality-filtered, and assessed using QIIME (version 1.17). Forward and reverse reads were merged using PEAR software (version v.0.9.5). Low-quality sequences shorter than 200 bp in length and with an average quality score less than 20 were removed prior to any additional analysis. Exact barcode matching was used, which allowed a two-nucleotide mismatch in primer matching. Reads containing ambiguous characters were also removed. The trimmed sequences were chimera checked, and those with chimeras were removed with the Uchime algorithm^75^. Only sequences that overlapped by more than 10 bp were assembled based on their overlapped sequences; reads that could not be assembled were discarded. The remaining and unique sequences were clustered at a 97% similarity using CD-HIT^76^ to yield operational taxonomic units (OTUs). To identify soil bacteria, Acidobacteria and fungi, and to estimate community diversity, the OTUs obtained were compared to the SILVA and UNITE databases.

When taxonomies were finished, the sequences that did not belong to Acidobacteria were removed before subsequent sequence analysis. To correct the sampling effort, the lowest sequencing number was randomly selected per sample and used for subsequent community analysis. All the bacterial sequences have been deposited in GenBank short-read archive under the following accession numbers: forest (SRR7287293, SRR7287316, SRR7287315), arable land (SRR7287330, SRR7287323, SRR7287324) and wetland (SRR7287307, SRR7287310, SRR7287309). All the Acidobacterial sequences have been deposited in GenBank short-read archive under the following accession numbers: forest (SRR7285667, SRR7285668, SRR7285669), arable land (SRR7285654, SRR7285657, SRR7285656) and wetland (SRR7285660, SRR7285653, SRR7285652). All fungal sequences have also been deposited in the GenBank short-read archive: forest (SRR7299284, SRR7299321, SRR7299322), arable land (SRR7299279, SRR7299303, SRR7299304) and wetland (SRR7299286, SRR7299328, SRR7299327).

### Statistical analysis

Alpha-diversity of the bacterial, Acidobacterial, and fungal communities included OTU richness, Chao1, and the Shannon-Wiener and Simpson’s indices, and the sequencing depth was expressed as the coverage calculated in MOTHUR. Estimates were calculated by employing the tools Aligner, Complete Linkage Clustering, and Rarefaction of the RDP pyrosequencing pipeline.

Beta-diversity was analyzed by PCoA using the Bray Curtis dissimilarity index. The selected OTUs had a similarity level of 0.03. This simple numeric visualization shows the similarity and overlap of the OTUs within all samples. Differences in the community structure (β-diversity) were assessed by conducting a permutational ANOVA (PERMANOVA, number of permutations = 99 999) with the Adonis function in the Vegan R package and displayed with PCoA ordinations. BioEnv and a canonical correspondence analysis (CCA) were performed to identify normalized OTU abundance and environmental variables that were most frequently related to bacterial, Acidobacterial, and fungal community structures in R using the Vegan package. Based on a diversity distance matrix of bacteria, Acidobacteria, and fungi, three dendrograms were constructed using cluster analysis and the unweighted pair-group method with arithmetic means (UPGMA) at the OTU level. All other statistical tests were performed in R software (v.2.8.1).

Soil physicochemical properties and alpha-diversity indices were analyzed using one-way analysis of variance (ANOVA) with a significance limit of P < 0.05. Relative abundances of all detected phyla were analyzed using one-way ANOVA with significance limits of P < 0.05 and P < 0.01. Least significant difference (LSD) procedures were used to determine significant differences between each habitat type. Standard deviation was calculated as the square root of the variance as a measure of the dispersion of a set of data from its mean.
